# Pirfenidone as a Cornerstone in the Management of Fibrotic Interstitial Lung Diseases and Its Emerging Applications: A Comprehensive Review

**DOI:** 10.7759/cureus.70497

**Published:** 2024-09-30

**Authors:** Harsh Babariya, Shilpa A Gaidhane, Sourya Acharya, Sunil Kumar

**Affiliations:** 1 Internal Medicine, Jawaharlal Nehru Medical College, Datta Meghe Institute of Higher Education and Research, Wardha, IND

**Keywords:** antifibrotic therapy, emerging applications, fibrotic interstitial lung diseases, idiopathic pulmonary fibrosis, pirfenidone, transforming growth factor-beta

## Abstract

Pirfenidone is a groundbreaking antifibrotic agent that has become a cornerstone in managing fibrotic interstitial lung diseases (ILDs), particularly idiopathic pulmonary fibrosis (IPF). This review comprehensively analyzes pirfenidone’s mechanisms of action, clinical efficacy, safety profile, and emerging applications beyond IPF. Pirfenidone exerts its therapeutic effects by inhibiting key pathways involved in fibrosis, including transforming growth factor-beta (TGF-β) and other pro-fibrotic cytokines. It also reduces oxidative stress and inflammation. Clinical trials have consistently demonstrated pirfenidone’s ability to slow the decline in lung function, reduce disease progression, and improve survival rates in IPF patients. Furthermore, emerging evidence supports its potential use in other fibrotic ILDs and non-pulmonary fibrotic conditions, such as liver and kidney fibrosis. Despite its proven benefits, pirfenidone’s safety and tolerability profiles require careful monitoring, with gastrointestinal and photosensitivity reactions being the most common adverse effects. Future research is poised to explore combination therapies, personalized treatment approaches, and novel applications of pirfenidone in a broader range of fibrotic disorders. As the field of antifibrotic therapy advances, pirfenidone remains a pivotal agent with the potential to significantly impact the management of fibrotic diseases across multiple organ systems. This review aims to provide clinicians and researchers with a detailed understanding of pirfenidone’s current role and prospects in treating fibrosis.

## Introduction and background

Fibrotic interstitial lung diseases (ILDs) encompass a diverse group of pulmonary disorders characterized by varying degrees of inflammation and fibrosis of the lung parenchyma [1]. These diseases include idiopathic pulmonary fibrosis (IPF), connective tissue disease-associated ILD (CTD-ILD), and chronic hypersensitivity pneumonitis (CHP), among others [2]. Despite their differing etiologies, these conditions share a common pathological feature: progressive scarring of lung tissue, which ultimately leads to compromised gas exchange and respiratory failure. This fibrosis-driven impairment in lung function manifests clinically as exertional dyspnea, a persistent non-productive cough, and, in advanced stages, hypoxemia and pulmonary hypertension [3]. The progressive nature of these diseases often results in a gradual decline in pulmonary function, typically measured by parameters such as forced vital capacity (FVC) and diffusing capacity of the lungs for carbon monoxide (DLCO). These changes not only severely impact patients’ quality of life but also contribute to increased morbidity and mortality, particularly in aggressive forms like IPF [4].

The pathophysiology of fibrosis in ILDs is complex and involves multiple interrelated processes. Initial injury to the alveolar epithelium - whether due to environmental exposures, autoimmune activity, or unknown factors - triggers an abnormal wound-healing response. Instead of normal tissue repair, a cascade of events leads to the activation of fibroblasts and their differentiation into myofibroblasts, cells responsible for excessive production of extracellular matrix (ECM), including collagen [5]. Central to this process is the dysregulation of several molecular pathways, with transforming growth factor-beta (TGF-β) playing a pivotal role. TGF-β promotes fibroblast proliferation and ECM deposition while inhibiting apoptosis of myofibroblasts, which would otherwise limit fibrosis. Additional factors, such as oxidative stress, epithelial-mesenchymal transition (EMT), and chronic inflammation, further exacerbate the fibrotic process. Over time, the accumulation of fibrotic tissue distorts lung architecture, leading to the formation of honeycomb cysts and obliteration of normal alveolar spaces [6].

Pirfenidone is an orally administered antifibrotic agent that has become a cornerstone in managing fibrotic ILDs, particularly IPF. The development of pirfenidone spans several decades, with its antifibrotic properties first identified in the 1970s [7]. Early studies demonstrated its efficacy in various animal fibrosis models, eventually leading to clinical trials in humans. Pirfenidone was first approved for the treatment of IPF in Japan in 2008, marking a significant milestone, as it became the first drug to receive such an indication [8]. Subsequent approvals followed in Europe (2011) and the United States (2014), supported by pivotal clinical trials that showed pirfenidone’s ability to slow the decline in lung function and reduce the risk of disease progression in IPF patients. Since its approval, pirfenidone has been the subject of extensive research, with growing interest in its potential applications beyond IPF, extending into other forms of fibrotic ILDs and even non-pulmonary fibrotic conditions [9].

The antifibrotic effects of pirfenidone are achieved through multiple mechanisms, making it a versatile agent in treating fibrosis. While the exact mechanisms of action are not fully understood, it is believed that pirfenidone inhibits key pro-fibrotic and pro-inflammatory pathways [10]. One of the primary mechanisms is the inhibition of TGF-β, a crucial cytokine that drives the fibrotic process by promoting fibroblast activation and ECM production. In addition to its effects on TGF-β, pirfenidone has been shown to reduce the production of other pro-fibrotic cytokines, such as tumor necrosis factor-alpha (TNF-α) and interleukin-1 beta (IL-1β) [11]. Furthermore, pirfenidone possesses antioxidant properties that help mitigate oxidative stress, a known contributor to fibrosis. By targeting these pathways, pirfenidone not only slows the progression of fibrosis but also modulates the inflammatory response that often accompanies fibrotic diseases [12]. This comprehensive review aims to provide an in-depth analysis of pirfenidone’s role in managing fibrotic ILDs, primarily focusing on IPF. Given the expanding use of pirfenidone in clinical practice, this review will also explore its emerging applications in other fibrotic conditions within and beyond the pulmonary system. By synthesizing current clinical evidence and ongoing research, the review offers valuable insights into the potential future directions of pirfenidone therapy.

## Review

Mechanism of action of pirfenidone

Pirfenidone exerts its therapeutic effects through antifibrotic, anti-inflammatory, and antioxidant properties. Although the precise mechanism of action is not fully understood, several key pathways have been identified that contribute to its efficacy in managing fibrotic diseases, particularly IPF [13]. A primary mechanism of pirfenidone is its anti-fibrotic effect: it reduces the production of TGF-β1, a crucial profibrotic cytokine involved in IPF pathogenesis. By inhibiting TGF-β1, pirfenidone prevents the differentiation of human lung fibroblasts into myofibroblasts, which are responsible for excessive collagen synthesis and ECM production [14]. This action limits fibroblast proliferation and significantly decreases collagen deposition in the lungs. Animal studies have demonstrated that pirfenidone effectively reduces biochemical and histopathological markers of fibrosis in various organs, including the lungs, liver, heart, and kidneys [15]. In addition to its antifibrotic properties, pirfenidone has notable anti-inflammatory effects. It modulates levels of pro-inflammatory cytokines, such as TNF-α, IL-1, IL-6, interferon-gamma (IFN-γ), and platelet-derived growth factor (PDGF) [15]. By downregulating these cytokines, pirfenidone helps to mitigate the inflammatory response associated with fibrotic diseases. Moreover, it promotes the production of the anti-inflammatory cytokine IL-10, enhancing its therapeutic profile. Pirfenidone also exhibits antioxidant actions by scavenging reactive oxygen species (ROS) and inhibiting lipid peroxidation, thereby reducing cellular injury and oxidative stress in the lungs [16]. Beyond its antifibrotic and anti-inflammatory actions, pirfenidone may affect additional pathways relevant to fibrosis. Research suggests it could inhibit angiogenesis and EMT, which play significant roles in the progression of IPF [17]. Additionally, pirfenidone has been shown to inhibit the formation and nuclear translocation of the transcriptional complex involving phospho-SMAD3, MUC1-CT, and active β-catenin, which are associated with TGF-β1 signaling. By reducing the activation of SMAD-binding elements, pirfenidone may further enhance its antifibrotic effects [18]. The detailed mechanisms of pirfenidone in fibrotic diseases are summarized in Table 1.

**Table 1:** Detailed mechanism of action of pirfenidone in fibrotic diseases

Mechanism	Description	Key pathways/targets	Impact on fibrotic process
Antifibrotic properties [19]	Pirfenidone inhibits pathways that promote fibrosis by targeting profibrotic factors, reducing fibroblast activation, and decreasing excessive collagen production	Inhibition of transforming growth factor-beta (TGF-β) signaling. Reduced activation of fibroblasts and myofibroblasts	Prevents the excessive deposition of extracellular matrix (ECM) proteins like collagen, reducing fibrosis progression
Anti-inflammatory effects [12]	Pirfenidone exhibits anti-inflammatory properties by modulating cytokine release and decreasing oxidative stress, thus limiting chronic inflammation in the lung environment	Suppression of pro-inflammatory cytokines (e.g., tumor necrosis factor-alpha (TNF-α), interleukin (IL)-1, IL-6). Inhibition of reactive oxygen species (ROS) production	Lowers inflammation-driven tissue damage, thereby reducing fibrosis-triggering inflammatory responses and cellular stress
Reduction in fibroblast proliferation [20]	Pirfenidone inhibits fibroblast proliferation and prevents their transformation into myofibroblasts, which are key cells responsible for the progression of tissue fibrosis	Direct inhibition of fibroblast growth and activity. Inhibition of platelet-derived growth factor (PDGF) signaling	Decreases fibroblast-induced fibrosis, slowing or halting the fibrotic remodeling of tissues
Inhibition of collagen synthesis [21]	It reduces the production of collagen, the primary component of fibrotic tissue, thereby mitigating the accumulation of scar tissue in affected organs	Decreased collagen gene expression. Inhibition of TGF-β1-mediated collagen synthesis	Prevents excessive collagen buildup, reducing the extent and progression of fibrosis
Inhibition of epithelial-mesenchymal transition (EMT) [22]	Pirfenidone reduces the conversion of epithelial cells into mesenchymal cells, a process involved in fibrosis progression by contributing to tissue remodeling	Inhibition of EMT markers (e.g., snail, twist and N-cadherin). Regulation of TGF-β-driven EMT pathways	Decreases the fibrotic transformation of epithelial cells, preventing the formation of fibrotic tissue in various organs
Impact on angiogenesis [23]	It modulates angiogenesis, which may indirectly contribute to fibrotic progression by influencing tissue oxygenation and remodeling processes	Inhibition of vascular endothelial growth factor (VEGF). Reduction in microvessel density	Reduces abnormal blood vessel formation associated with tissue remodeling in fibrosis, contributing to a more stable tissue structure
Reduction of oxidative stress [24]	Pirfenidone has antioxidant properties, reducing oxidative stress, which is known to contribute to cellular injury and fibrosis	Scavenging of free radicals. Inhibition of oxidative stress pathways, including nicotinamide adenine dinucleotide phosphate (NADPH) oxidase	Limits oxidative stress-induced damage to lung cells, preventing the exacerbation of fibrotic changes
Reduction in cellular stress responses [25]	By reducing endoplasmic reticulum (ER) stress and apoptosis, pirfenidone protects cells from stress-induced damage that contributes to the development and progression of fibrosis	Decreased ER stress markers. Inhibition of stress-induced apoptosis pathways	Alleviates cellular stress responses, preventing apoptosis and further tissue remodeling associated with fibrosis

Clinical evidence in fibrotic ILDs

Pirfenidone has been evaluated in several pivotal clinical trials, including the CAPACITY trials (studies 004 and 006) and the ASCEND trial. The CAPACITY 004 study demonstrated that patients receiving pirfenidone experienced a slower decline in FVC over a 72-week treatment period. However, the CAPACITY 006 study did not replicate this benefit over the same duration [26]. In contrast, the ASCEND trial reported significant improvements in FVC decline, increases in six-minute walk test distances, and enhanced progression-free survival during the 52-week follow-up period. Collectively, these trials underscore the efficacy of pirfenidone in managing IPF [27]. Long-term efficacy and safety data from pooled analyses of the CAPACITY and ASCEND trials further confirm the benefits of pirfenidone in slowing disease progression in IPF over a year. The treatment was associated with a notable reduction in the risk of all-cause mortality, FVC decline, hospitalization, and an increase in exercise capacity. Generally, pirfenidone is well tolerated, with the most common adverse events being gastrointestinal and skin-related, typically mild to moderate in severity [27]. Pirfenidone's impact on survival and quality of life is substantial. A pre-specified analysis of the pooled data from the CAPACITY and ASCEND trials indicated that pirfenidone reduced the risk of death by 48% in one year compared to placebo. Additionally, the risk of treatment-emergent death due to IPF at one year was reduced by 68% in the pirfenidone group. This significant reduction in mortality, coupled with improvements in lung function and exercise capacity, underscores the drug's importance in enhancing the quality of life for patients with IPF [26,27]. For non-IPF fibrotic ILDs, the evidence for pirfenidone's efficacy in CTD-ILD is still emerging. While specific data on pirfenidone's effectiveness in CTD-ILD are limited, a Cochrane review suggested that it improves progression-free survival and, to a lesser extent, pulmonary function in patients with IPF. This implies potential applicability in CTD-ILD, though further studies are needed to establish its role definitively [28]. Emerging data also suggest that pirfenidone may be effective in treating CHP and unclassifiable progressive fibrosing ILDs. However, more research is required to validate these findings and understand the full spectrum of pirfenidone's efficacy in various fibrotic lung diseases. Additionally, there are no head-to-head trials comparing pirfenidone and nintedanib in IPF or other fibrotic ILDs. Both drugs have shown efficacy in slowing disease progression in IPF, but their relative effectiveness in non-IPF fibrotic ILDs remains uncertain [29]. Observational studies and registries have documented real-world experience with pirfenidone, confirming its benefits in mitigating FVC declines in IPF, consistent with clinical trial data. These studies offer valuable insights into the drug's effectiveness in routine clinical practice. However, further research is needed to comprehensively assess long-term outcomes, patient adherence, and tolerability of pirfenidone in everyday settings [8].

Emerging applications beyond fibrotic ILDs

Pirfenidone is being investigated for its potential applications in various pulmonary conditions beyond fibrotic ILDs. One area of interest is its use during acute exacerbations of ILDs. Although specific studies on this application are currently limited, the antifibrotic properties of pirfenidone may offer therapeutic benefits by mitigating further lung damage during these critical episodes [30]. Additionally, given the rising incidence of pulmonary fibrosis following COVID-19, researchers are exploring pirfenidone’s potential to prevent or treat post-COVID-19 pulmonary fibrosis. Preliminary studies suggest that its anti-inflammatory and antifibrotic effects could be beneficial in this context, though more extensive clinical trials are needed to confirm its efficacy [31]. Beyond pulmonary conditions, pirfenidone shows promise in treating liver fibrosis and chronic kidney disease (CKD). Clinical trials have indicated that pirfenidone can slow the progression of renal function decline in patients with CKD, suggesting a systemic antifibrotic effect [32]. Additionally, studies have demonstrated its potential efficacy in liver fibrosis, particularly in conditions such as chronic hepatitis and non-alcoholic steatohepatitis. However, further research is necessary to establish definitive benefits in these areas. Investigational use in cardiac fibrosis and systemic sclerosis is also gaining attention. Animal models have shown that pirfenidone can reduce cardiac fibrosis, associated with improved heart function. In systemic sclerosis, its anti-fibrotic properties may help manage skin and organ fibrosis, although clinical data remain preliminary [32]. The potential for combination therapies involving pirfenidone is another promising area of research. There is growing interest in the synergistic effects of pirfenidone when combined with other antifibrotic or anti-inflammatory agents [33]. This approach may enhance therapeutic outcomes in fibrotic diseases by targeting multiple pathways involved in fibrosis. Several clinical trials are currently investigating the efficacy of pirfenidone in combination with other treatments for various fibrotic conditions. These trials aim to determine optimal dosing strategies and the potential for improved patient outcomes through combination therapy [33]. Emerging applications of pirfenidone beyond fibrotic ILDs are summarized in Table 2.

**Table 2:** Emerging applications of pirfenidone beyond fibrotic interstitial lung diseases

Emerging application	Condition	Potential role	Current evidence/studies
Other pulmonary conditions [34]	Acute exacerbations of interstitial lung diseases (ILDs)	Potential to reduce progression and severity of exacerbations	Preliminary evidence from small clinical studies
	Post-COVID-19 pulmonary fibrosis	May help prevent or mitigate fibrosis post-COVID-19 infection	Early studies and case reports, ongoing research
Non-pulmonary applications [35]	Liver fibrosis	Antifibrotic effects in reducing hepatic scarring	Animal models and small-scale human studies
	Chronic kidney disease (CKD)	Potential role in slowing progression of kidney fibrosis	Investigational use in CKD, preclinical and early-phase trials
	Cardiac fibrosis	Potential to reduce cardiac fibrosis, improving heart function	Investigational, limited clinical data
	Systemic sclerosis	Role in mitigating skin and organ fibrosis	Emerging evidence, clinical trials in progress
Combination therapies [36]	With other antifibrotic agents	Synergistic effects in improving efficacy, reducing fibrosis	Ongoing trials exploring combinations with nintedanib or others
	With anti-inflammatory agents	Potential to enhance anti-inflammatory benefits and reduce damage	Studies investigating combination with corticosteroids or biologics
	With novel agents	Exploration of combinations with emerging therapies	Early research and experimental models

Safety and tolerability

Pirfenidone is generally well-tolerated; though, like any medication, it carries a risk of adverse effects. Understanding its safety profile and implementing risk mitigation strategies are crucial for optimizing patient outcomes [8]. Common side effects of pirfenidone are typically mild to moderate in severity. Gastrointestinal disturbances, such as nausea, diarrhea, anorexia, and abdominal pain, often occur early in treatment but may improve over time. Photosensitivity, characterized by skin reactions like rashes or sunburn-like symptoms due to increased sensitivity to sunlight, is also common. Patients are advised to use sunscreen and protective clothing when outdoors. Fatigue is frequently reported, which can impact quality of life [7]. Serious adverse events are rare but can occur and require prompt attention. Elevations in liver enzymes (alanine aminotransferase (ALT) and aspartate aminotransferase (AST)) have been noted, necessitating regular monitoring of liver function tests. Rarely, patients may experience severe skin reactions, such as Stevens-Johnson syndrome or toxic epidermal necrolysis. There have also been infrequent reports of acute exacerbations of ILDs or pneumonia, which require immediate evaluation and management [37]. Effective management of pirfenidone therapy involves proactive strategies to minimize risks and enhance tolerability. The recommended starting dose is 267 mg thrice daily, though this may be adjusted based on individual tolerance. Liver enzymes should be monitored at baseline and periodically to detect abnormalities early. Dose adjustments may be necessary for significant gastrointestinal side effects, and gradual dose titration can help improve tolerability [38]. Patient education is key. Patients should be informed about potential side effects, the importance of reporting unusual symptoms, and measures to mitigate photosensitivity, such as sun protection. Providing access to support groups or counseling can help patients manage the emotional and psychological aspects of living with a chronic illness and dealing with treatment side effects. Regular follow-up visits are essential for ongoing treatment efficacy and tolerability assessment, as well as timely intervention if adverse effects arise [39]. Understanding pirfenidone's safety and tolerability is crucial for effective management of patients with fibrotic ILDs. By recognizing common side effects, monitoring for serious adverse events, and implementing risk mitigation strategies, healthcare providers can enhance patient safety and improve overall treatment outcomes [8].

Future directions in pirfenidone research

Research into pirfenidone is expanding rapidly beyond its established use in IPF to explore its potential in treating various non-pulmonary fibrotic conditions. Current clinical trials investigate its efficacy in systemic sclerosis and liver fibrosis. These studies aim to determine the drug's role in managing a broader range of fibrotic diseases, potentially offering new therapeutic options for patients with limited alternatives [14]. In addition to exploring new indications, researchers focus on novel formulations and delivery methods for pirfenidone. Innovative drug delivery systems, such as inhalable formulations using nanoparticles and liposomes, are being developed to enhance the drug's effectiveness. These advanced formulations aim to improve the targeting of the lungs while reducing systemic side effects commonly associated with oral administration, such as gastrointestinal disturbances. Such advancements could lead to more effective and patient-friendly treatment options [40]. The concept of personalized medicine is gaining traction in the context of pirfenidone therapy. One promising approach is integrating biomarker-guided therapy in ILDs. Identifying specific biomarkers could enable healthcare providers to tailor treatment protocols, optimizing the efficacy of pirfenidone while minimizing adverse effects. This approach enhances treatment outcomes and aligns with the growing trend toward individualized patient care [41]. Furthermore, pharmacogenomics is a critical area of research focusing on how genetic variations influence patient responses to pirfenidone. By examining genetic profiles, researchers aim to develop more personalized treatment regimens that cater to each patient's unique characteristics. This could significantly improve the overall effectiveness of pirfenidone therapy, ensuring that patients receive the most appropriate and effective treatment based on their genetic makeup [42]. The future of pirfenidone research also includes exploring its potential role in combination with gene therapies or regenerative medicine approaches. Investigating the synergistic effects of pirfenidone in conjunction with these innovative treatments could enhance its antifibrotic properties and contribute to tissue regeneration in fibrotic diseases. Such combination therapies could significantly advance the management of chronic fibrotic conditions, offering hope for improved outcomes [7]. Moreover, researchers are exploring the expansion of pirfenidone's indications to include rare fibrotic disorders, which often lack established treatment options. Clinical trials are being designed to assess the safety and efficacy of pirfenidone in these conditions, potentially broadening the therapeutic landscape for patients suffering from various forms of fibrosis. As research progresses, pirfenidone may emerge as a versatile treatment option for IPF and a wide array of fibrotic diseases, thereby enhancing patient care and outcomes [32]. Emerging research directions for pirfenidone in fibrotic diseases are summarized in Table 3.

**Table 3:** Emerging research directions for pirfenidone in fibrotic diseases

Category	Research focus	Details
Ongoing and upcoming clinical trials [13]	Trials targeting non-pulmonary fibrotic conditions	Research into pirfenidone's role in liver fibrosis, chronic kidney disease, and cardiac fibrosis
Investigations into novel formulations [43]	Exploring new delivery methods	Studies on sustained-release formulations or inhaled versions for improved patient compliance
Combination therapies [44]	Synergistic effects with other agents	Investigations into combining pirfenidone with nintedanib, corticosteroids, or immune modulators
Personalized medicine [41]	Biomarker-guided therapy in interstitial lung diseases (ILDs)	Use of biomarkers to predict response to pirfenidone and tailor treatments for better efficacy
Pharmacogenomics [45]	Patient-specific considerations	Genetic research to understand patient variability in response to pirfenidone and minimize side effects
Gene therapy [46]	Potential role of pirfenidone in gene-based therapies	Exploring pirfenidone in combination with gene-editing technologies to treat fibrotic disorders
Regenerative medicine [47]	Use in combination with stem cell or regenerative treatments	Investigating the potential of pirfenidone to support tissue regeneration in fibrotic diseases
Expanding indications [48]	Research into rare fibrotic disorders	Exploring the use of pirfenidone in diseases like systemic sclerosis, sarcoidosis, and other rare ILDs

## Conclusions

In conclusion, pirfenidone has established itself as a pivotal therapy in the management of fibrotic ILDs, particularly IPF. Its multifaceted mechanisms of action, including inhibiting key pro-fibrotic and pro-inflammatory pathways, have been instrumental in slowing disease progression and improving patient outcomes. The clinical efficacy of pirfenidone, supported by robust trial data and real-world experience, underscores its value as a cornerstone treatment in IPF. Additionally, emerging evidence suggests potential applications of pirfenidone beyond IPF in other fibrotic conditions within and outside the pulmonary system, highlighting its versatility and broad therapeutic potential. However, while pirfenidone has shown promise, its safety and tolerability profiles necessitate careful patient management, and ongoing research is essential to further refine its use. As the landscape of antifibrotic therapy continues to evolve, pirfenidone remains a key player, with future directions likely to focus on combination therapies, personalized medicine approaches, and expanding indications to address unmet needs in fibrotic diseases.
